# PTD-FNK Alleviated LPS-Induced Oxidative Stress of Boar Testicular Sertoli Cells via Keap1-Nrf2 Pathway

**DOI:** 10.3390/vetsci11110543

**Published:** 2024-11-06

**Authors:** Weixia Ji, Qiuyan Huang, Qiqi Ma, Xingxing Song, Xin Zhang, Xun Li, Xiaoye Wang, Sutian Wang, Yanling Wang, Zhengzhong Xiao, Chuanhuo Hu

**Affiliations:** 1 College of Animal Science and Technology, Guangxi Key Laboratory of Animal Breeding, Disease Control and Prevention, Guangxi University, Nanning 530004, China; 2 State Key Laboratory of Swine and Poultry Breeding Industry, Guangdong Key Laboratory of Animal Breeding and Nutrition, Institute of Animal Science, Guangdong Academy of Agricultural Sciences, Guangzhou 510640, China; 3 Animal Husbandry Research Institute, Guangxi Vocational University of Agriculture, Nanning 530001, China

**Keywords:** PTD-FNK, Sertoli cell, oxidative stress, *Nrf2* pathway

## Abstract

PTD-FNK, a synthetic anti-apoptotic protein, has been shown to potently alleviate cellular injuries. However, the effects of PTD-FNK on oxidative defense in boar testicular Sertoli cells (SCs) against oxidative injury has not been explored. In this study, we show that exposure of SCs to 100 mg/L lipopolysaccharide (LPS) for 12 h leads to decreased survival rate, superoxide dismutase (SOD) activity, and increased malondialdehyde (MDA). Treatment with 0.01 nmol/L PTD-FNK for 4 h significantly enhanced the activity of SOD, catalase (CAT), glutathione peroxidase (GSH-Px), and total antioxidant capacity (T-AOC) in SCs. Concurrently, PTD-FNK treatment effectively reduced the production of reactive oxygen species (ROS) and the levels of 8-hydroxy-2′-deoxyguanosine (8-OHdG) in SCs. Moreover, using His pull-down and LC-MS techniques, we identified PTD-FNK-interacting proteins and confirmed that this protective effect may be mediated by the regulation of the *Keap1*-*Nrf2* signaling pathway by PTD-FNK. Therefore, PTD-FNK alleviates LPS-induced oxidative stress via the *Keap1*/*Nrf2* pathway, providing novel insights for the development of therapeutic agents targeting testicular oxidative damage.

## Simple Summary

This study investigated the protective effects of the anti-apoptotic protein PTD-FNK on SCs under oxidative stress induced by LPS. We found that PTD-FNK treatment enhanced antioxidant enzyme activities and reduced ROS production and oxidative damage markers in SCs. Furthermore, PTD-FNK was shown to potentially regulate the *Keap1-Nrf2* signaling pathway, suggesting its therapeutic potential for testicular oxidative damage.

## 1. Introduction

Oxidative stress (OS) is recognized as a significant factor contributing to the development of various health conditions, including reproductive disorders, in both animal and human studies [1]. The SCs in the reproductive system are the main cells targeted by various toxins, making them an excellent cellular model for studying damage to the male animal reproductive system in vitro. SCs play a vital role by supplying essential growth factors and chemokines needed for germ cell development [2]. The proliferation of SCs is a key factor influencing the population of mature SCs, which in turn affects spermatogenesis in adult male animals. Additionally, SCs directly regulate the release of the blood–testis barrier (BTB) and various immunomodulators [1]. Hence, any factor that disrupts its normal development can lead to abnormal sperm development, ultimately resulting in male infertility [3,4]. During spermatogenesis, sperm are released from the lacunae of SCs into the proximal compartment, while SCs also phagocytose shed sperm cell bodies and degenerative spermatogenic cells. This process leads to the absorption of substances by SCs, resulting in the production of ROS such as superoxide anions (O2-) and hydroxyl radicals (OH-). Disruption of the redox balance in SCs impairs their ability to repair DNA damage, leading to chromosomal structural abnormalities, gene-expression disorders, and cellular apoptosis and contributing to various diseases and reduced survival rates in young animals. Additionally, external stimuli can trigger excessive ROS production in SCs. The strong antioxidant function of SCs involves enhancing the activity of SOD and GSH [5].

The *Keap1*/*Nrf2* pathway is one of the most crucial antioxidant signaling pathways within cells, playing a pivotal role in maintaining cellular homeostasis in response to oxidative stress. When the body is subjected to oxidative stress, an anti-oxidative system is activated as a defense mechanism. The *Keap1*/*Nrf2* pathway is essential in this balance. Studies have indicated that under hypoxic conditions, miR-141 can modulate the *Keap1*/*Nrf2* signaling pathway to prevent oxidative stress-induced damage [6], thereby reducing apoptosis and enhancing cell survival rates. Hepatoprotective substances (HPSs) ameliorate liver oxidative stress injury through the *Keap1*/*Nrf2* pathway [7,8]. Additionally, coumarin has been shown to protect against hydrogen peroxide-induced oxidative damage in human ovarian granulosa cells by regulating the *Keap1*/*Nrf2*/*ARE* signaling pathway [9,10]. Therefore, the maintenance of the normal *Keap1*/*Nrf2* pathway function is of great significance for preserving cellular homeostasis and preventing the onset of diseases. This research highlights the potential therapeutic targets for the treatment of oxidative stress-related pathologies.

The FNK protein, a synthetic cell-protection protein derived from the rat BCL-xL protein [11], has been shown in studies to confer increased resistance to oxidative damage, heat stress, apoptotic processes, and other stimuli in cells expressing high protein levels. The FNK protein is fused with a membrane-active protein-transduction domain (PTD), forming PTD-FNK; the PTD consists of 11 amino acid residues with the sequence YGRKKRRQRRR; the protein PTD-FNK gains the capability to traverse cell membranes, enabling it to facilitate the entry of large molecules and drugs, as well as other exogenous substances, into cells and effectively cross the blood–brain barrier [12,13,14]. Moreover, the activity of PTD-FNK is maintained through chemical cross-linking or genetic-engineering techniques [13,15,16,17]. This feature effectively protects against cell apoptosis and necrosis triggered by diverse harmful stimuli [14].

Moreover, research has shown that PTD-FNK possesses significant cytoprotective functions. It serves as a protective agent capable of reducing cell death during the processes of freezing and thawing. Additionally, PTD-FNK can prevent necrosis and acute liver injury induced by CCL4 [11,18]. Importantly, it can rapidly repair ischemic brain injury through its fast transduction properties within a short time frame. It also can effectively decrease cell death induced by LPS, improve frozen sperm viability, and preserve sperm mitochondrial integrity [19,20]. It displays a remarkable ability to protect against various forms of cellular damage and stimulation, indicating potential for valuable clinical applications.

To date, there is a lack of research on the impact of PTD-FNK on the oxidative damage of SCs. Therefore, the main aim of this study was to explore how PTD-FNK alleviates LPS-induced oxidative damage in boar SCs and establish a theoretical basis for its potential application in animal reproductive studies.

## 2. Materials and Methods

### 2.1. Testis Collection

SCs were isolated from the testes of two-week-old piglets, disinfected and stripped of the tunica albuginea. The testicular tissue was minced into an emulsion and treated with 2 mg/mL collagenase IV for 30 min at 37 °C. The digestion was terminated by centrifugation at 1000 rpm for 5 min. The pellet was washed twice with PBS and treated with 5 mg/mL DNase I for 20 min at 37 °C. An equal volume of complete culture medium was added to halt the digestion. The mixture was filtered successively through 200-mesh and 400-mesh sieves, followed by centrifugation at 1000 rpm for 5 min. After two additional washes with PBS, the pellet was resuspended and treated with red blood cell lysis buffer for 5 min at room temperature to lyse any residual erythrocytes. The cells were then washed three times with PBS by centrifugation. The cells were subsequently seeded into T25 flasks and incubated at 37 °C in a humidified atmosphere containing 5% CO_2_.

All experiments were conducted in accordance with the guidelines of the local Animal Ethics Committee and approved by the Ethics Committee of Guangxi University. Testes for SC isolation were obtained from healthy piglets aged two weeks at a suburban pig farm in Nanning, Guangxi.

### 2.2. Experimental Design

The aim of this study was to assess the protective effect of PTD-FNK against LPS-induced oxidative damage in porcine SCs. Initially, an oxidative stress model was established using porcine SCs. Subsequently, the cells were cultured in 96-well plates with 200 μL of serum-free medium per well. Different concentrations of LPS (0, 0.01, 0.1, 1, 10, and 100 mg/L) in serum-free DMEM/F12 were added for various durations (0, 4, 8, 16, and 24 h). Cell viability as well as SOD and MDA levels were measured to determine the optimal concentration and duration for inducing oxidative damage in SCs by LPS. To identify the optimal concentration and duration for PTD-FNK action, cells of each subgroup were cultured in a serum-free medium with different concentrations of PTD-FNK (0.01, 0.1, 1, 10, 100 nmol/L) for different periods (0.5, 1, 2, 4, 8 h); cell counting kit-8 (CCK8) assay was used to determine cell viability to screen the optimal concentration and time of PTD-FNK incubation.

### 2.3. Oil Red O Stain Analysis and Identification

Cells at a density of 1 × 10^5^ cells/mL were seeded in 6-well plates and cultured until reaching 100% confluence. Subsequently, the cells were washed with PBS on both sides and stained with Oil Red O staining reagent following the instructions (Cat.G1262, Solarbio). Oil Red O staining was performed on the SC samples. Randomly selected areas were observed under a microscope to examine the presence of red lipid droplets around the cytoplasmic poles or nuclei of the cells. The marker genes *GATA4* and *SOX9* of SCs were used for PCR verification, and the sequences are shown in Table 1.

### 2.4. Cell Counting Kit-8 (CCK8)

The cells at a 1 × 10^4^ cells/mL density were seeded in 96-well plates and incubated in a 37 °C CO_2_ incubator. Subsequently, 10 μL of CCK8 solution (Cat.CK04, DOJINDO, Tokyo, Japan) was added to each well, followed by a 2 h incubation in the dark. Each well’s optical density (OD) was measured at 450 nm using a microplate reader, providing an indirect assessment of the viability of boar SCs.

### 2.5. Reactive Oxygen (ROS) Species Quantification in SCs

SCs were quantitatively assessed for ROS levels using a Reactive Oxygen Species assay kit (E004-1-1, Jiancheng, Nanjing, China). Cells at a concentration of 1 × 10^6^ cells/mL were seeded into a 6-well plate and cultured in a 37 °C CO_2_ incubator until reaching 80% confluence. After 24 h of corresponding treatment, cells were incubated with ROS assay reagent at 37 °C for 30 min, followed by fluorescence intensity measurement using flow cytometry(Cytoflex LX; Elementar, UK).

### 2.6. Measurement of Oxidant-Antioxidant Status in Boar SCs

The levels of GSH-Px, T-AOC, CAT and 8-OHdG (Cat.A005-1-2, Cat.A015-1-2, Cat.A007-1-1, Cat.H165-1-2, Jiancheng, Nanjing, China) in SCs were measured strictly following the manufacturer’s instructions. All tests were conducted in triplicates to ensure accuracy.

### 2.7. Real-Time Quantitative PCR (qRT-PCR)

For quantitative analysis, total RNA was isolated from SCs using Trizol Reagent (Cat.R411, Vazyme). cDNA was synthesized using All-In-One 5× RT Master Mix (Cat.R211, Vazyme). qRT-PCR was conducted using Taq Pro Universal SYBR qPCR Master Mix (Cat.Q712, Vazyme) with the following PCR program: 95 °C for 30 s, 95 °C for 5 s, (62 °C for 30 s, 72 °C for 30 s) for 30 cycles, and 72 °C for 5 min. The 2^−∆∆Ct^ values were determined post-amplification. qRT-PCR primers for genes SOD2, GSH-px, CAT, HSPA5, VIM, *Nrf2*, *Keap1*, *NQO1*, and *HO-1* were synthesized by Qingke Biotechnology Co., Ltd., and designed using NCBI’s Primer-Blast program. The housekeeping gene *β-actin* was used as an internal reference control. The primer list used is provided in Table 2.

### 2.8. Enzyme-Linked Immunosorbent Analysis (ELISA)

Nrf2 and Keap1 levels were quantified in the cell culture supernatant using an enzyme-linked immunosorbent assay (ELISA). The supernatants were collected, and the assays were conducted according to the manufacturer’s instructions (KIGENE, Shanghai, China). Following the termination of the reaction, the optical density (OD) values were determined at a wavelength of 450 nm.

### 2.9. Western Blot

The expression levels of Nrf2, Keap1, NQO1, and HO-1 in SCs were evaluated using Western blot analysis. After gently washing the cells with PBS, total cellular proteins were extracted by adding RIPA lysis buffer containing a mixture of protease/phosphatase inhibitors. The supernatants were collected by centrifugation at 12,000× *g* at 4 °C for 10 min. The protein concentration of each sample was detected using a BCA assay kit (Abcam, Cambridge, UK). SDS-PAGE electrophoresis was performed after adding loading buffer to the protein extractions and boiling. After transferring the proteins onto a PVDF membrane, the membrane was blocked with 5% skim milk for 1 h. The membrane was subsequently incubated with corresponding Nrf2, Keap1, NQO1, *β-actin* and HO-1(1:1000) antibodies overnight at 4 °C on a shaker, washed with TBST three times, followed by secondary antibody at room temperature for 1 h. After extensive washing with TBST, the membrane was subjected to ECL chemiluminescence analysis and image capture. The relative expression levels of each protein were normalized with *β-actin* using ImageJ software (National Institutes of Health; version 1.45).

### 2.10. His Pull-Down

The PTD-FNK interaction protein was separated when passing through Ni beads by making Ni beads and combining His label, and then the separated bands were analyzed by mass spectrometry. The polypeptide sample was diluted in a solution of 2% acetonitrile and 0.1% formic acid before being analyzed using a Triple TOF 5600 plus mass spectrometer and an Eksigent nanoLC system (AB SCIEX, Framingham, MA, USA). ProteinPilot was used to analyze the original mass spectrometry MS/MS file. According to the results of the identified protein, a certain filtration standard was selected, the unused score > 1.3 was considered a reliable peptide, and the protein containing at least one unique peptide was retained.

### 2.11. Data Analysis

The data were presented as means ± standard deviation (SD) and represented three independent experiments. GraphPad Prism 10.2.2 was utilized for mapping. ANOVA was applied for statistical analysis using SPSS23.0 software. Statistical significance was indicated by an asterisk in the figures (ns, *p* > 0.05; *, *p* < 0.05; **, *p* < 0.01; ***, *p* < 0.001; **** *p* < 0.0001).

## 3. Result

### 3.1. Boar SC Isolation

**Figure 1:**
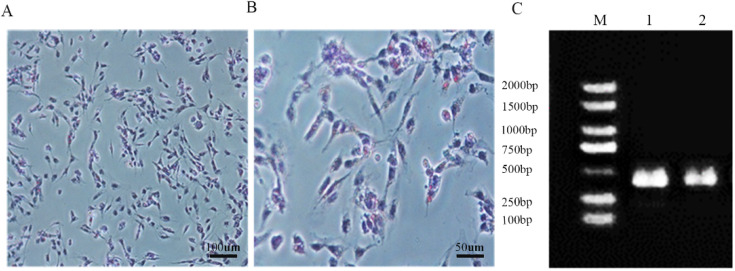
(**A**,**B**) The morphology of SCs was identified by Oil Red O staining; (**C**) arrows indicate the bipolar corpuscles in the nucleus. The SC marker gene was detected by PCR. 1.*GATA4*; 2.*SOX9*; M.DL2000 Marker.

To establish an oxidative injury model in SCs, we initially isolated SCs from healthy testes. The morphology of isolated SCs was evaluated using Oil Red O staining. The results showed that the nuclei were stained with blue-purple, while the cytoplasm was lighter and reddish. Red vacuolar fat droplets can be observed at the two cytoplasm poles or around the nucleus (Figure 1A,B). Meanwhile, ovoid or irregular nuclei at two to three can be observed in the atypical cells, exhibiting multiple protrusions. To further confirm the isolation of SCs, we detected the well-documented marker genes of SCs. The results showed that the cells express high SC marker genes GATA binding protein 4(*GATA4*) and SRY-box transcription factor 9(*SOX9*) (Figure 1C). Collectively, these results suggest that the SCs were successfully isolated.

### 3.2. The Establishment of an Oxidative Stress Model

LPS impacted the proliferation of SCs. The OD values decreased significantly in all dose groups with increasing LPS concentrations compared to the control group, indicating reduced viable cells. Additionally, all groups showed lower OD values than the control group (*p* < 0.05). Specifically, after exposure to 100 mg/L LPS for 12, 16, and 24 h, the cell OD values were recorded as 0.50 ± 0.003, 0.47 ± 0.003, and 0.43 ± 0.010, respectively, with statistically significant differences (*p* < 0.05). This result suggests that at an LPS concentration of 100 mg/L and a treatment duration of 12 h, there was a higher likelihood of oxidative damage occurrence (*p* < 0.05) (Table S1). The SOD content exhibited a dose-dependent decrease in the various LPS groups compared to the control groups, with statistically significant variances (*p* < 0.05). Except for the treatment group exposed to 0.01 mg/L LPS for 4 h (*p* > 0.05), there were no significant differences between the control group and the other treatment groups (Table S2).

Moreover, there was a significant dose-dependent increase in malondialdehyde (MDA) content in all LPS groups compared to the control group, indicating a notable escalation in oxidative damage. These results affirm the successful establishment of the oxidative stress model, particularly evident in the substantial induction of oxidative damage to SCs after 12 h of exposure to 100 mg/L LPS (Table S3).

### 3.3. Effect of PTD-FNK on the Activity of SCs

The viability of SCs in every PTD-FNK dose group exhibited a significant increase compared to the control group. The highest cell viability observed after a 4 h treatment with 0.01 nmol/L PTD-FNK was particularly noteworthy, highlighting its superior protective efficacy on SCs (*p* < 0.05). These findings underscore the substantial enhancement in the activity of LPS-induced SCs conferred by PTD-FNK under the 0.01 nmol/L (Table S4).

### 3.4. Effect of PTD-FNK on the Antioxidant Capacity of SCs

**Figure 2:**
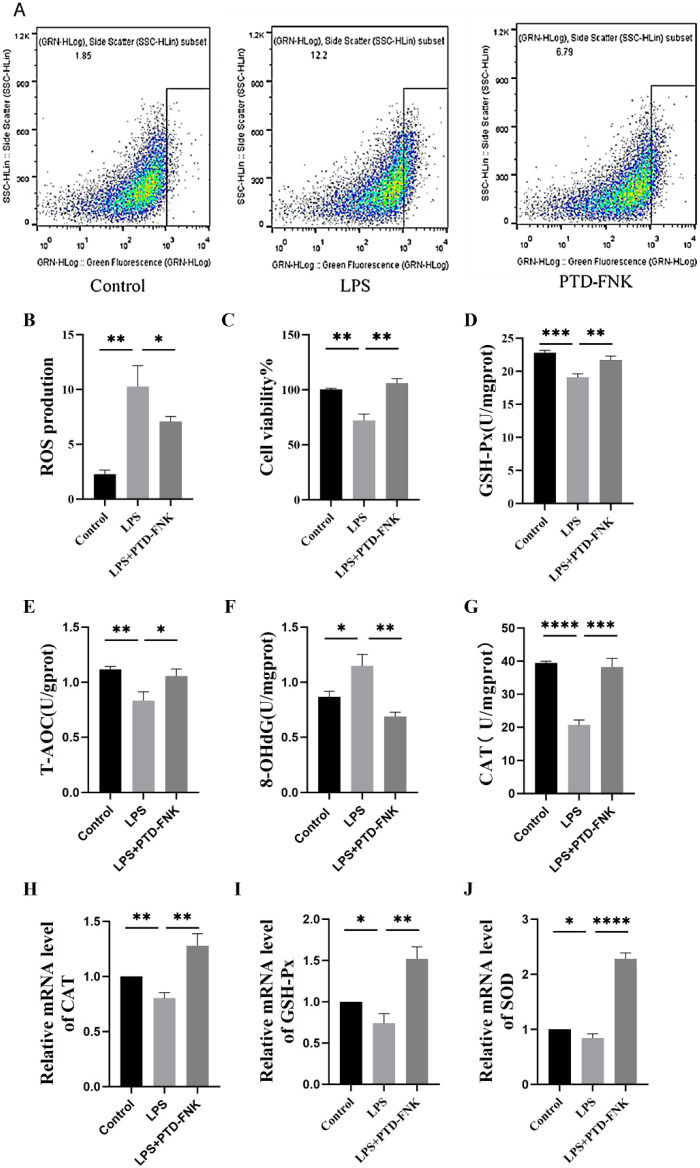
(**A**,**B**) Effects of PTD-FNK on ROS production of LPS-induced boar SCs; (**C**) CCK8 assays of the PTD-FNK effect on cell viability; (**D**–**G**) effects of PTD-FNK on GSH-Px, CAT, 8-OHdG, and T-AOC contents of LPS-induced boar SCs; (**H**–**J**) effects of PTD-FNK on mRNA expression of GSH-Px, CAT, and SOD in LPS-induced boar SCs. *n* ≥ 3 (* *p* < 0.05; ** *p* < 0.01; *** *p* <0.001; **** *p* < 0.0001).

SCs were treated with 100 mg/L LPS for 12 h, followed by treatment with 0.01 nmol/L PTD-FNK for 4 h. The fluorescence intensity of ROS in SCs was measured at 10.20 ± 2 in the LPS group, 6.79 ± 1.03 in the PTD-FNK group, and 1.85 ± 0.75 in the control group (Figure 2A). A significant increase in ROS levels was observed in the LPS group compared to the control group (*p* < 0.01). Additionally, a significant decrease in ROS fluorescence intensity was noted in the PTD-FNK group compared to the LPS group (*p* < 0.05) (Figure 2B). PTD-FNK is capable of restoring cellular viability at the cellular level (*p* < 0.05) (Figure 2C).

**Figure 3:**
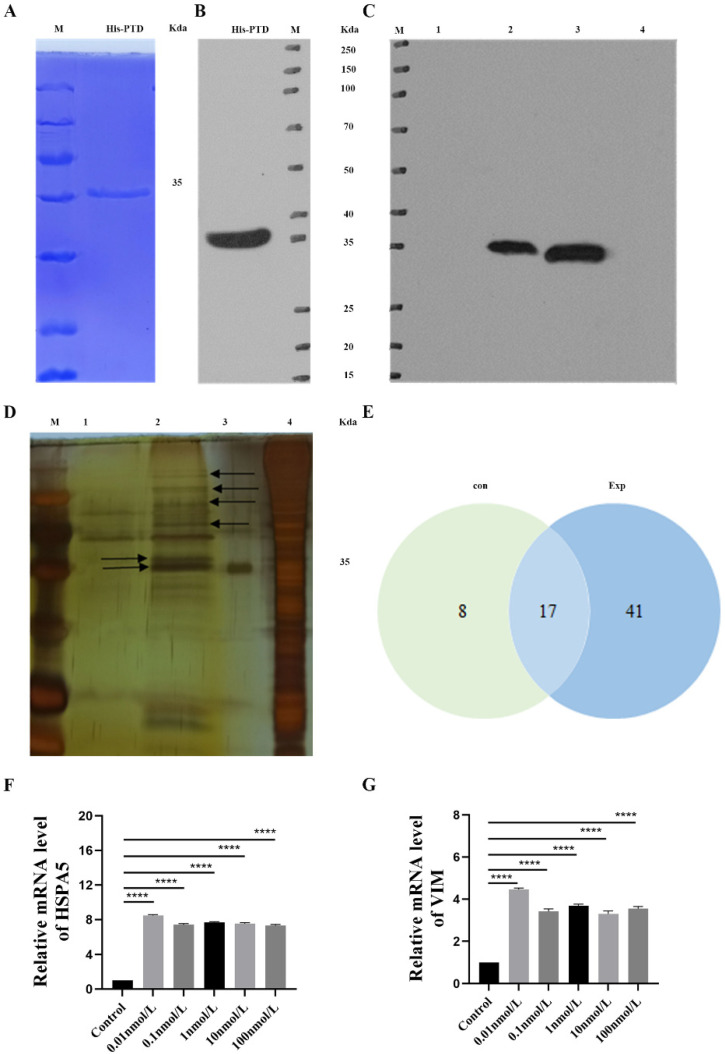
(**A**) His recombinant protein was detected by CBB; (**B**) His recombinant protein was detected by WB; (**C**) the expression of SCs His antibody was detected by WB; (**D**) “silver stain” detection result after pull-down; the red arrow represents the bait protein; the black arrows indicate the PTD-FNK interacting proteins; (**E**) Venn diagram of PTD-FNK differential protein sets in boar SCs; (**F**,**G**) PTD-FNK effects on *HSPA5* and *VIM* mRNA expression in boar SCs. *n* ≥ 3 ****p < 0.0001).

In comparison to the control group, the levels of GSH-Px, T-AOC (*p* < 0.001), and CAT (*p* < 0.05) were reduced in the LPS group (Figure 3D–E,G), while the levels of 8-OHdG (*p* < 0.05) were elevated (Figure 2F). Conversely, treatment with PTD-FNK resulted in an up-regulation of GSH-Px (*p* < 0.001), T-AOC (*p* < 0.01), and CAT (*p* < 0.05), along with a down-regulation of 8-OHdG (*p* < 0.001) compared to the LPS group.

Furthermore, in comparison to the control group, the mRNA expressions of CAT (*p* < 0.01), GSH-Px (*p* < 0.05), and SOD (*p* < 0.05) (Figure 2H–J) were down-regulated in the LPS group. Conversely, compared to the LPS group, the LPS+PTD-FNK group exhibited an up-regulation of mRNA expressions for CAT (*p* < 0.01), GSH-Px (*p* < 0.001), and SOD (*p* < 0.001).

These results indicate that PTD-FNK can enhance the antioxidant capacity of SCs to mitigate the oxidative damage induced by LPS.

### 3.5. His Pull-Down Analysis of PTD-FNK Interaction Protein

In the Coomassie brilliant blue method (CBB) and WB analyses, a single signal corresponding to the size of the target protein (35 Kda) was detected, confirming that the PTD-FNK protein carried a His label and was suitable for further experiments (Figure 3A,B). The WB and silver stain detection results from the pull-down assay demonstrated successful extraction of the PTD-FNK interaction protein, allowing for further protein quality analysis via mass spectrometry (Figure 3C,D).

Mass spectrometry analyzed the differences in proteins between the two groups of samples. A total of 66 proteins were identified, with 25 proteins in the control group, 58 proteins in the experimental group with PTD-FNK protein, and 17 proteins detected in both groups (Figure 3E). High-ranking proteins included HSPA5, vimentin, histone, myosin, actin, villin-1, keratin, and tubulin β chain (Table 3). For differential protein-specific information, see Table S5.

Compared to the control group, treatment with 0.01, 0.1, 1, 10, and 100 nmol/L of PTD-FNK increased expression levels of and VIM mRNA (Figure 3F,G). The most significant enhancement was observed at a PTD-FNK concentration of 0.01 nmol/L, consistent with the mass spectrometry analysis. These findings indicate that PTD-FNK mainly binds to the HSPA5 protein and participates in the Nrf2 antioxidant pathway.

### 3.6. PTD-FNK to Activate the Nrf2/Keap1 Pathway in LPS-Induced Boar SCs

**Figure 4:**
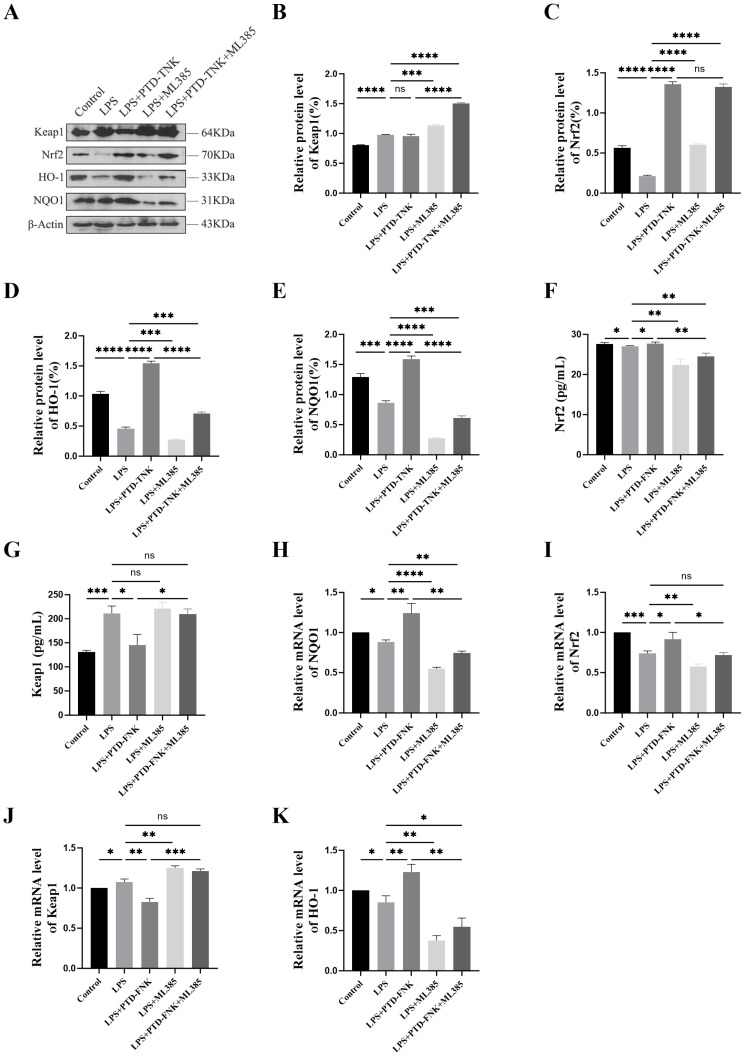
(**A**–**E**) The relative protein level of Nrf2, Keap1, HO-1, and *NQO1*; (**F**–**G**) Nrf2 and Keap1 protein levels were measured using ELISA; (**H**–**K**) *Nrf2*, *Keap1*, *HO-1*, and *NQO1* mRNA levels; control, no drug added; LPS, induced by 100 mg/L LPS for 12 h; LPS + PTD-FNK, protected by 0.01 mmol/L PTD-FNK for 4 h and then induced by 100 mg/L LPS for 12 h; LPS+ML385, induced by 5μM ML385 for 2 h, followed induced by 100 mg/L LPS for 12 h; LPS + PTD-FNK + ML385, induced by 5μM ML385 for 2 h, protected by 0.01 mmol /L PTD-FNK for 4 h, and then induced by 100 mg/L LPS for 12 h. *n* ≥ 3 (* *p* < 0.05; ** *p* < 0.01; *** *p* < 0.001; **** *p* < 0.0001).

The expressions of NQO1, HO-1, Nrf2, and Keap1 proteins were assessed using Western blot analysis. In comparison to the control group, LPS treatment resulted in an up-regulation of Keap1 protein content (*p* < 0.01), while the protein levels of Nrf2 (*p* < 0.05), *HO-1* (*p* < 0.001), and *NQO1* (*p* < 0.05) were decreased. Furthermore, when comparing the PTD-FNK group to the LPS group, it was observed that PTD-FNK down-regulated Keap1 protein expression (*P* < 0.01) and significantly up-regulated Nrf2 (*p* < 0.01), HO-1 (*p* < 0.001), and *NQO1* (*p* < 0.05) protein expression. These results indicate that PTD-FNK may counteract the effects of LPS-induced oxidative stress in SCs by modulating the expression of key antioxidant proteins (Figure 4A,E).

To delve deeper into the mechanisms underlying the antagonistic effects of PTD-FNK in LPS-stimulated SCs, we employed the Nrf2 inhibitor, 5 μM ML385 (HY-100523,MCE) for subsequent experiments. ELISA was used to determine serum Nrf2 and Keap1 protein levels. In the LPS group, Nrf2 protein concentration decreased while Keap1 protein expression was enhanced (*p* < 0.01) (Figure 4F,G). Additionally, the LPS group exhibited increased *Keap1* mRNA expression (*p* < 0.001) alongside decreased levels of *Nrf2* (*p* < 0.01), *HO-1* (*p* < 0.05), and *NQO1* (*p* < 0.05) (Figure 4H–K). Interestingly, the addition of ML385 mimicked the effects of oxidant stress. Our findings revealed that ML385 effectively inhibited Nrf2 activity and the regulatory effects of PTD-FNK on oxidative damage (*p* < 0.05). These results suggest that PTD-FNK may alleviate oxidative stress severity by modulating the Nrf2 pathway.

## 4. Discussion

This study has elucidated two key findings. Our results demonstrate that PTD-FNK effectively mitigates the oxidative damage induced by LPS in SCs. Secondly, PTD-FNK modulates the Keap1-Nrf2 pathways to reduce oxidative damage and apoptosis. Notably, the administration of ML385, an Nrf2 inhibitor, negated the positive regulatory effects of PTD-FNK on the response to oxidative stress.

In our experiment, we isolated SCs and successfully identified them by Oil Red O staining and the marker genes *GATA4* and *SOX9* [21,22]. SCs are essential for testicular development during embryogenesis and for mature spermatogenesis by governing the microenvironment surrounding maturing germ cells [23]. LPS is an endotoxin that can induce damage both in vitro and in vivo. It can stimulate cells to produce a large amount of ROS, thereby leading to apoptosis. Some studies have demonstrated the production of LPS-induced reactive oxygen species intermediates and lipid peroxides [24,25]. Our study demonstrates that treatment with 100 mg/L LPS significantly reduced the expression of antioxidant enzymes in porcine SCs, such as SOD and MDA. The expression of ROS increased, leading to a substantial decline in cell viability. Subsequent rescue with PTD-FNK significantly reduced ROS levels, and cell viability was markedly restored. In the study investigating the effects of the PTD-FNK protein on lipopolysaccharide-induced acute lung injury in rats, it was found that PTD-FNK enhanced the viability of alveolar epithelial cells in a dose-dependent manner in vitro [20]. Prolonged treatment with PTD-FNK led to a gradual decline in cell viability, which aligns with our findings. In our study, treating LPS-stimulated SCs with PTD-FNK at a concentration of 100 nmol/L for 8 h significantly enhanced the viability of SCs. This indicates that PTD-FNK effectively reverses the excessive ROS production induced by LPS exposure and restores the antioxidant capacity of SCs. Kazuki’s research [19] also confirmed the presence of the PTD-FNK protein and demonstrated its protective effect against sperm apoptosis during cryopreservation caused by mitochondrial dysfunction. Asoh’s work [26] provided additional insight by showing that PTD-FNK rapidly penetrates cells and targets mitochondria, a critical observation given that mitochondria are the primary site of ROS production, and their dysfunction can result in ROS buildup. Therefore, applying PTD-FNK may reduce ROS production in SCs, safeguarding mitochondrial function.

Mass spectrometry is widely employed in scientific research, and in this study, we identified proteins interacting with PTD-FNK, including HSPA5, VIM, ACTB, and VIL1 through mass spectrometry experiments. HSPA5 plays a crucial role in oxidative stress responses; during endoplasmic reticulum (ER) stress, HSPA5 expression is upregulated, and it binds to misfolded or unfolded proteins to restore protein homeostasis [27,28,29]. Concurrently, it triggers the oligomerization of PERK and IRE1, direct substrates of *Nrf2*, thereby exerting a regulatory effect. Studies have shown that HSPA5 inhibits the nuclear translocation of ROS and *Nrf2* in DLD-1 colon cancer cells, indicating a regulatory role for HSPA5 in cellular responses to oxidative stress [30]. VIM is a structural protein in SCs that is essential for maintaining their distinctive shape, facilitating sperm maturation and ejaculation, and preserving the integrity of the blood–testis barrier (BTB) [31]. ACTB is a major component of the microfilaments in the cytoskeleton and is involved in various physiological functions of cells [32]. VIL1 affects cell morphology and function by regulating the microfilament network. When oxidative stress occurs, the cellular homeostasis is disrupted, which can lead to apoptosis under severe conditions [33]. PTD-FNK can inhibit the release of the apoptotic protein cytochrome C (CytC) from the mitochondria, thus preventing apoptosis [34]. Further analysis and validation of genes interacting with PTD-FNK protein were carried out. The qRT-PCR analysis uncovered a substantial upregulation of *HSPA5* and *VIM* mRNA following PTD-FNK treatment. This result suggests that PTD-FNK can engage with *HSPA5* and *VIM* in SCs, thereby reinforcing the structural robustness and functional capacity of these cells and strengthening their ability to withstand stressors.

To comprehensively understand how PTD-FNK safeguards against LPS-induced damage to SCs, we directed our investigation towards the *Keap1*/*Nrf2* signaling pathway. This pathway is recognized for its vital role in countering oxidative stress by eliminating reactive oxygen species [35,36]. Central to this antioxidant defense system is *Nrf2*, a key transcription factor that ordinarily binds to *Keap1*, forming an inactive complex. *Keap1* is sensitive to changes in the intracellular redox environment. When oxidative stress is detected, *Nrf2* is released from *Keap1*, triggering the transcription of genes encoding a variety of phase II detoxifying enzymes and antioxidant enzymes, thereby bolstering the body’s overall antioxidant defense. The data revealed a substantial elevation in the protein levels and mRNA expression of *Keap1* after exposure to LPS, coupled with a significant decline in the mRNA expression of *HO-1*, *Nrf2*, and *NQO1*. These observations are in agreement with some findings [37,38,39]. Our research showed that PTD-FNK significantly enhanced the expression of both Nrf2 protein and mRNA while reducing the expression of Keap1 protein and mRNA. However, the administration of ML385 resulted in a pronounced decrease in the expression of proteins and mRNA related to the *Nrf2*-*Keap1* pathway, thus reversing the protective effects of PTD-FNK against oxidative stress. These findings indicate that PTD-FNK may exert its protective effects in SCs against oxidative damage by regulating the *Keap1*/*Nrf2* pathway. The primary objective of this study was to determine the effect of PTD-FNK on LPS-induced activation of SC cells. Numerous studies have indicated that LPS can influence crucial physiological functions of cells by binding to the TLR4 receptor and secreting inflammatory factors such as TNF-α, IL-1, and IL-6, leading to an inflammatory response in the body. LPS has also been shown to increase the incidence of early miscarriage in mice, which may be due to a reduction in progesterone (P4) production, preventing the uterus from contracting normally and resulting in implantation failure and miscarriage [40]. Another possible reason is that LPS may interfere with fertility by affecting oocytes [41]; studies have shown that LPS stimulation inhibits the maturation of oocytes in mice and cattle, thereby affecting fertility [42,43]. Additionally, research has found that LPS can mediate an inhibitory effect on the reproductive axis by suppressing the secretion of GnRH and LH, which is an important factor affecting fertility [44]. Although this study did not verify the effects of LPS on inflammatory factors and related hormones and did not confirm whether PTD-FNK can mitigate these effects, the results of this experiment provide a promising direction. The findings suggest that PTD-FNK could be applied in research related to anti-inflammatory effects and hormone secretion.

## 5. Conclusions

In summary, the treatment with 0.01 nmol/L PTD-FNK effectively combats LPS-induced oxidative injury in porcine SCs, potentiating their structural and functional robustness through regulating *HSPA5* and *VIM*, thus alleviating stress responses. These protective effects’ mechanisms are linked to PTD-FNK’s ability to modulate the *Keap1*-*Nrf2* signaling pathway.

## Figures and Tables

**Table 1:** List of primer sequences for PCR.

Primer Name	Base Sequence(5′ to 3′)	Length	Accession Number
*GATA4*	F: 5′-TCTCGATATGTTTGATGAACTTCTC-3′ R: 5′-GTCTTCGATTTGTTA AGGTTCTTG-3′	378	XM_013990299.2
*SOX9*	F:5′- CCGGCTCCTACTACAGCCAC-3′ R:5′- GTGGCCAGGCCACTCTTGCT-3′	383	NM_213843.2

**Table 2:** List of primer sequences for qRT-PCR.

Primer Name	Base Sequence (5′ to 3′)	Length	Accession Number
*β-actin*	F:5′-CTAGTTACACACACGCGGCTCT-3′ R:5′-CATGAATACCCTGCACAGATCG-3′	127	XM_003357
*SOD2*	F: 5′-CTGGCCAAGGGAGATGTTAC-3′ R: 5′-AAAGACCCAAAGTCACGCTT-3′	167	NM_001322819.2
*GSH-px*	F:5′-CTCATGACCGACCCCAAGTT-3′ R:5′-GTCAGAAAGCGACGGCTGTA-3′	128	NM_214201.1
*CAT*	F: 5′-CCTGCAACGTTCTGTAAGGC-3′ R: 5′-ATATCAGGTTTCTGCGCGGC-3′	109	NM_214301.2
*Nrf2*	F: 5′-GACTCCAAGGGGTTGCGAAGG-3′ R: 5′-CCCAAACCCCAATCCCGTAG-3′	80	XM_005671981.3
*Keap1*	F: 5′-CATCGGCATCGCCAACTTC-3′ R: 5′-ACCAGTTGGCAGTGGGACAG-3′	135	NM_012289.4
*NQO1*	F:5′- ATGTATGACAAAGGACCCTTCC -3′ R:5′ - TCCCTTGCAGAGAGTACATGG -3′	88	NM_001159613.1
*HSPA5*	F: 5′-CATCACGCCGTCCTATGTCG-3′ R: 5′-CGTCAAAGACCGTTCTCG -3′	104	NM_005347.5
*VIM*	F:5′-CAGTATGAAAGCGTGGCTGC-3′ R:5′-AGGGACTCGTTAGTGCCTTT-3′	197	NM_003380.5
*HO-1*	F: 5′-TGTACCGCTCCCGAATGAAC-3′ R: 5′-TGGTCCTTAGTGTCCTGGGT-3′	142	NM_001004027.1

F: Forward; R: Reverse.

**Table 3:** Protein information table of PTD-FNK interaction in porcine SCs.

Accession	Name	Gene	Coverage	Unique Peptides
A0A4X1UFV5	GRP78	*HSPA5*	51	4
A0A4X1UCE3	Vimentin	*VIM*	31	15
A0A4X1TQB9	Actin	*ACTB*	37	14
F1SRY3	Villin-1	*VIL1*	8	6
F1SGG3	Cytokeratin-1	*KRT1*	8	5
A0A5G2QGK	Tubulin beta chain	*TUBB4B*	14	1
A0A4X1TY33	Tropomyosin alpha-1 chain	*TPM1*	12	4
A5A759	Keratin 2A	*KRT2A*	5	2
A0A4X1VYY1	Prelamin-A/C	*LMNA*	7	4

## Data Availability

No new data was created.

## Supplementary Materials

The following supporting information can be downloaded at: https://www.mdpi.com/article/10.3390/vetsci11110543/s1, Table S1. Effects of LPS on proliferation of boar SCs; Table S2. The effect of LPS on SOD (U/mgprot) content in SCs; Table S3. Effects of LPS on MDA (nmol/mgprot) content in SCs; Table S4. Effect of PTD-FNK on the activity of SCs; Table S5. An Interaction Profile of PTD-FNK Protein in SCs.
