# Supplementary material for: PTD-FNK Alleviated LPS-Induced Oxidative Stress of Boar Testicular Sertoli Cells via Keap1-Nrf2 Pathway
Source: Vet Sci. 2024 Nov 6;11(11):543. doi: 10.3390/vetsci11110543 (PMC11598942; doi:10.3390/vetsci11110543)
Supplement: Supplementary file 1 [file vetsci-11-00543-s001.zip › vetsci-3174174-supplementary.pdf]

Supplementary Materials

PTD-FNK Alleviated LPS-Induced Oxidative Stress of Boar Testicular Sertoli Cells via Keap1-Nrf2 Pathway

Weixia Ji [1,†], Qiuyan Huang [1,2,†], Qiqi Ma [1], Xingxing Song [1], Xin Zhang [1], Xun Li [1], Xiaoye Wang [1], Sutian Wang [2], Yanling Wang [3,*], Zhengzhong Xiao [3,*] and Chuanhuo Hu [1,*]

Table S1. Effects of LPS on proliferation of boar SCs.

	4 h	8 h	12 h	16 h	24 h
Control	0.70±0.001^{a}	0.70±0.003^{a}	0.68±0.001^{a}	0.68±0.002^{a}	0.66±0.002^{a}
0.01 mg/L	0.70±0.001^{a}	0.67±0.003^{b}	0.66±0.001^{b}	0.63±0.003^{b}	0.61±0.002^{b}
0.1mg/L	0.70±0.002^{a}	0.65±0.004^{d}	0.64±0.002^{c}	0.62±0.002^{c}	0.60±0.002^{c}
1mg/L	0.71±0.013^{a}	0.66±0.002^{c}	0.64±0.002^{d}	0.61±0.005^{d}	0.59±0.003^{c}
10mg/L	0.71±0.006^{a}	0.66±0.010^{cd}	0.62±0 003^{e}	0.48±0.002^{e}	0.46±0.001^{d}
100 mg/L	0.65±0.003^{b}	0.65±0.001^{d}	0.50±0.003^{f}	0.47±0.003^{f}	0.43±0.010^{e}

Data are expressed as the mean ± standard error. (*n*=3). Values in the same column with different superscripts are significantly different, while values with the same superscripts are not significantly different. (The same below).

Table S2. The effect of LPS on SOD (U/mgprot) content in SCs.

	4 h	8 h	12 h	16 h	24 h
Control	21.50±0.038^{a}	22.57±0.240^{a}	21.33±0.233^{a}	21.86±0.188^{a}	21.55±0.109^{a}
0.01 mg/L	17.30±0.193^{b}	16.80±0.263^{b}	16.04±0.379^{b}	14.93±0.334^{b}	14.33±0.220^{b}
0.1 mg/L	16.65±0.400^{c}	16.26±0.242^{c}	14.73±0.146^{b}	14.34±0.206^{c}	13.87±0.095^{b}
1 mg/L	16.50±0.211^{c}	15.72±0.138^{d}	13.63±0.130^{c}	13.30±0.163^{d}	13.00±0.131^{c}
10 mg/L	15.93±0.283^{d}	15.37±0.205^{d}	13.39±0.313^{c}	12.76±0.133^{e}	12.56±0.253^{c}
100 mg/L	15.73±0.166^{d}	14.83±0.141^{e}	12.85±0.100^{d}	12.56±0.091^{e}	12.06±0.094^{d}

Table S3. Effects of LPS on MDA(nmol/mgprot) content in SCs.

	4 h	8 h	12 h	16 h	24 h
Control	0.85±0.003^{d}	0.63±0.003^{e}	0.62±0.001^{f}	0.72±0.001^{f}	0.73±0.008^{f}
0.01 mg/L	0.95±0.09^{d}	1.08±0.11^{d}	1.29±0.05^{e}	1.04±0.015^{e}	1.25±0.005^{e}
0.1 mg/L	1.12±0.023^{c}	1.22±0.165^{d}	1.54±0.03^{d}	1.34±0.01^{d}	1.27±0.02^{d}
1 mg/L	1.66±0.045^{b}	1.86±0.01^{c}	1.93±0.04^{c}	1.93±0.04^{c}	2.06±0.036^{c}
10 mg/L	1.81±0.032^{a}	1.92±0.012^{b}	2.13±0.1^{b}	2.16±0.03^{b}	2.17±0.042^{b}
100 mg/L	1.89±0.045^{a}	2.07±0.033^{a}	2.34±0.01^{a}	2.43±0.045^{a}	2.48±0.021^{a}

Table S4. Effect of PTD-FNK on the activity of SCs.

	0.5h	1h	2h	4h	8h
Control	0.49±0006^{c}	0.48±0.004^{c}	0.47±0.004^{d}	0.47±0.002^{c}	0.47±0.002^{c}
0.01 nmol/L	0.51±0.009^{a}	0.52±0.003^{b}	0.71±0.012^{c}	1.14±0.009^{a}	0.80±0.007^{b}
0.1 nmol/L	0.53±0.005^{a}	0.53±0.004^{b}	0.72±0.001^{c}	0.96±0.008^{c}	0.88±0.008^{a}
1 nmol/L	0.51±0.004^{b}	0.53±0.002^{a}	0.80±0.003^{b}	0.98±0.002^{b}	0.89±0.014^{a}
10 nmol/L	0.52±0.003^{a}	0.54±0.004^{a}	0.82±0.002^{a}	0.95±0.004^{d}	0.92±0.038^{a}
100 nmol/L	0.52±0.006^{a}	0.55±0.012^{a}	0.81±0.004^{ad}	0.94±0.004^{d}	0.92±0.014^{a}

Table S5. An Interaction Profile of PTD-FNK Protein in Porcine SCs.

Accession	Gene	Coverage	Unique peptides
A0A4X1UFV5	*HSPA5*	51	4
A0A4X1UCE3	*VIM*	31	15
A0A4X1TQB9	*ACTB*	37	14
F1SRY3	*VIL1*	8	6
F1SGG3	*KRT1*	8	5
A0A5G2QGK	*TUBB4B*	14	1
A0A4X1TY33	*PM1*	12	4
A5A759	*KRT2A*	5	2
A0A4X1VYY1	*LMNA*	7	4
